# EFFECT OF AN INTENSIVE LIFESTYLE INTERVENTION IN ADULTS WITH TYPE 2 DIABETES ON LONG-TERM HEALTH CARE SPENDING AND USE

**DOI:** 10.1093/geroni/igad104.1701

**Published:** 2023-12-21

**Authors:** Ian Davis, Christopher Frenier, Peter Huckfeldt, Ann Harada, Nicholas Pajewski, Mark Espeland, Dana Goldman

**Affiliations:** University of Southern California, Los Angeles, California, United States; Yale University, New Haven, Connecticut, United States; University of Minnesota School of Public Health, Minneapolis, Minnesota, United States; Leonard D. Schaeffer Center for Health Policy & Economics, Los Angeles, California, United States; Wake Forest University School of Medicine, Winston-Salem, North Carolina, United States; Wake Forest University School of Medicine, Winston-Salem, North Carolina, United States; University of Southern California, Los Angeles, California, United States

## Abstract

This study investigates the impact of the Look AHEAD intensive lifestyle intervention (ILI) on long-term health care spending. We investigated self-reported health care use and spending from 2002-2012 (during the intervention) and 2013-2019 (after the intervention). Lasso regressions, trained in Medicare data, were used to predict outpatient, inpatient, prescription drug, and total healthcare spending based on self-reported health care use. Other outcomes included self-reported outpatient visits, unique prescription drugs, and inpatient hospitalizations. Regressions comparing spending and use outcomes for the ILI and diabetes support and education (DSE) control groups were adjusted for baseline demographic characteristics, clinical status, and study site. We included 3957 participants (1997 ILI, 1960 DSE) who had at least one interview during the post-intervention period. Similar to prior work, we found that the ILI group had lower inpatient, outpatient, and prescription drug spending relative to the DSE group during the intervention period, resulting in lower total spending (adjusted difference: -$715: [95% CI: -$1178 to -$252]; P<0.001). However, most of the intervention’s effects on spending and use did not persist beyond the intervention period, except for prescription drug spending (adjusted difference: -$189: [95% CI: -$364 to -$14]; P=0.03) and unique prescriptions (adjusted difference: -0.3: [95% CI: -0.5 to -0.1]; P<0.001). Our results imply that an intensive lifestyle intervention can reduce health care spending for adults with type 2 diabetes, but interventions may need to be continued to maintain spending reductions.

